# Secondary conditions in adolescents and young adults (AYA) with spina bifida (SB) in Four US Programs

**DOI:** 10.1186/1743-8454-7-S1-S22

**Published:** 2010-12-15

**Authors:** Timothy J Brei, Kathleen J Sawin, Constance Buran, Thomas Webb, Susan E Cashin, Amy Heffelfinger

**Affiliations:** 1 School of Medicine, Developmental Medicine Department, Indiana University, Indianapolis, USA; 2 College of Nursing and Children’s Hospital of Wisconsin, UW-Milwaukee, Box 413, Milwaukee, Wi 53201, USA; 3 Ambulatory Administration, Riley Hospital for Children, 702 Barnhill Dr, ROC 3205 Indianapolis, IN 46202, USA; 4 Huntersville Pediatrics and Internal Medicine, Presbyterian Novant Medical Group, 17220 Northcross Dr., Huntersville, NC 28036, USA; 5 College of Health Sciences, University of Wisconsin-Milwaukee, Box 413, Milwaukee, USA; 6 Department of Neurology, Division of Neuropsychology, Medical College of Wisconsin, 9200 W. Wisconsin, Ave, Milwaukee, Wi 53226-3596, USA

## Background

Spina bifida affects one out of every 1200 to 1400 live births each year in the United States. Secondary conditions related to the level of the SB lesion (LOL) such as incontinence, skin breakdown, obesity, pain, and orthopedic problems (e.g., scoliosis) are common. The purpose of this presentation is to address the following questions: 1) What is the frequency of key secondary conditions (bowel/bladder status, skin breakdown, UTIs, pain, overweight, scoliosis, latex allergy)? 2) Are select secondary conditions related to LOL, school, peer or employment activities? 3) How satisfied are participants with their bowel/bladder program and is satisfaction related to clinical variables?

## Materials and methods

134 parents/ youth pairs from 4 clinical programs participated in telephone interviews. Data were collected using parent and youth “Demographic and Clinical Information Form” (DCIF), the Functional Independence Measure (FIM™), and review of the medical records.

## Results

The parent sample was predominantly Caucasian. 62% had private insurance, with 28% reporting Medicaid and 11% reporting SSI for secondary income and 50% had an annual income of $50,000 or more. AYA were aged 12-25, M=15.5 (SD=3.24), 48% were male. LOL was: 32% thoracic/high lumbar, 25% lumbar, 14% lumbar/sacral and 9% other or unclear. 40% of AYA walked with or without crutches, 7% walked and used a wheelchair for sports/distance, 42% used a manual chair and 11% used a power chair for ambulation. Eighty-seven percent of the AYA had a shunt with a mean revision rate = 2.63, range (0-15); 37% were hospitalized in the last year. A substantial number of participants reported secondary conditions in the past year: skin breakdown (43%); overweight (37%), pain (47%); or scoliosis (40%). 64% had UTIs in past 3 years. 19-41% of participants reported these secondary conditions interfered with school, job or recreation with pain having the highest interference and overweight the least. The average AYA needs some assistance with bladder/bowel program and has accidents (1-2 or less a month). Parent and AYA satisfaction with bladder program was not associated with amount of assistance needed nor LOL but was related to number of bladder accidents (r=.39; .29 respectively). The same pattern was found with satisfaction with bowel program.

## Conclusions

Parents report a substantial number of AYA with at least one secondary condition. Pain appears to interfere most with activities and overweight the least. Prevention and effective treatment of secondary physical conditions are important for full participation in society.

